# Head growth trajectories as a window into neurodevelopment in preterm infants^☆^

**DOI:** 10.1016/j.jped.2024.07.001

**Published:** 2024-07-09

**Authors:** Thiviya Selvanathan

**Affiliations:** a Department of Pediatrics, University of British Columbia and BC Children's Hospital Research Institute, Vancouver, Canada; b Department of Pediatrics, The Hospital for Sick Children, Toronto, Canada

Although there have been significant advances in neonatal intensive care over the last few decades, preterm infants remain at increased risk for neurodevelopmental problems that persist into adulthood.^1^, ^2^, ^3^ Children born preterm have altered brain maturation, or brain dysmaturation, which includes smaller total and regional brain volumes, altered white matter microstructural maturation, and impaired brain connectivity.^4^ For example, in a study of children born preterm followed to adolescence, slower brain growth and smaller brain volumes were observed in children born preterm compared to children born at term.^5^ Brain dysmaturation in children born preterm has been associated with neurodevelopmental impairments.^4^

Head circumference measurements may provide a window into assessing brain maturation in preterm infants in the clinical setting. Previous studies have shown that head circumference measurements are related to total cerebral volumes in preterm infants.^6^^,^^7^ In a cohort of preterm infants with neurodevelopmental follow-up to school-age, a small head circumference at birth was associated with poorer neurodevelopmental outcomes at school-age. However, catch-up growth with normalization of small birth head circumference prior to discharge from the NICU was associated with better outcomes.^6^ Other studies have also observed associations between postnatal head circumference catch-up growth and improved neurodevelopmental outcomes in children born preterm, although findings have varied across cohorts.^8^, ^9^, ^10^, ^11^

Mayrink et al.^12^ followed a prospective cohort of very preterm infants without a history of major neonatal critical illness by performing serial head circumference measurements and neurodevelopmental assessments from birth to two years of age. There was an overall decrease in head circumference growth during the period of neonatal intensive care with a decrease in z scores from birth to discharge. After discharge from hospital, there was a period of accelerated head growth to 1 month corrected age following which head circumference z scores remained stable. Interestingly, with serial neurodevelopmental assessments performed at 12, 18 and 24 months, there was an increase in neurodevelopmental delays detected in this cohort across timepoints. Larger head circumference at 5 months was associated with higher motor, cognitive and language scores at 18 months corrected age.

The findings of Mayrink et al.^12^ reinforce the importance of monitoring head circumference growth in children born preterm. Head circumference measurements can be performed easily in any clinical setting, including those with limited resources and limited access to neuroimaging. Monitoring head circumference trajectories could allow for earlier identification of children who are at increased risk for neurodevelopmental impairments, which may result in implementation of early neurodevelopmental supports and interventions. Specifically, their findings, and those of others, suggest that failure of head circumference catch-up growth in early infancy may be potential marker of an increased risk for neurodevelopmental concerns, although optimal timing of measurements remains unknown.^6^^,^^8^^,^^10^, ^11^, ^12^

The increased prevalence of neurodevelopmental concerns observed over time in the study by Mayrink et al.^12^ highlight the importance of longitudinal follow-up to assess neurodevelopmental trajectories in children born preterm. They found an increased prevalence of neurodevelopmental impairments at 24 months compared to 18 months corrected age in their cohort suggesting that later age of assessment may be important for identifying more subtle neurodevelopmental concerns. Their findings are similar to those in a large cohort of very preterm infants with higher odds of a diagnosis of significant developmental impairments in infants who were assessed at 21–24 months compared to children who were assessed at 18–20 months corrected age.^13^ Further, although the authors followed their cohort to 2 years of age, studies examining associations with long-term neurodevelopmental outcomes into childhood and adolescence are important as more subtle impairments may become apparent as children become older.^14^ Future studies should also consider assessing neurodevelopmental *trajectories* over time rather than outcomes at a single timepoint.^15^ Finally, although the authors excluded preterm infants with major neonatal critical illness in their study and appropriately addressed this as a limitation of their work, future studies of associations between head growth and neurodevelopmental trajectories should also include infants born preterm with major neonatal morbidities so that findings are generalizable to clinical populations of preterm infants.

## Conflicts of interest

The author declares no conflicts of interest.
